# Short Chain Fatty Acids Taken at Time of Thrombectomy in Acute Ischemic Stroke Patients Are Independent of Stroke Severity But Associated With Inflammatory Markers and Worse Symptoms at Discharge

**DOI:** 10.3389/fimmu.2021.797302

**Published:** 2022-01-19

**Authors:** Nicholas Henry, Jacqueline Frank, Christopher McLouth, Amanda L. Trout, Andrew Morris, Jianzhong Chen, Ann M. Stowe, Justin F. Fraser, Keith Pennypacker

**Affiliations:** ^1^ Department of Neurology, University of Kentucky, Lexington, KY, United States; ^2^ Center for Advanced Translational Stroke Science, University of Kentucky, Lexington, KY, United States; ^3^ Department of Behavioral Science, University of Kentucky, Lexington, KY, United States; ^4^ Department of Neurosurgery, University of Kentucky, Lexington, KY, United States; ^5^ Division of Cardiovascular Medicine, University of Kentucky, and Lexington Veterans Affairs Healthcare System, Lexington, KY, United States; ^6^ Oligonucleotide Bioanalysis Research - Chemistry, Dicerna Pharmaceuticals Inc., Lexington, MA, United States; ^7^ Department of Neuroscience, University of Kentucky, Lexington, KY, United States; ^8^ Department of Radiology, University of Kentucky, Lexington, KY, United States

**Keywords:** inflammation, brain ischemia, microbiome & dysbiosis, cytokine - immunological terms, neuroimmunology of the gut

## Abstract

**Introduction:**

Short chain fatty acids (SCFA) are gut microbiota-derived metabolites that contribute to the gut-brain axis and may impact stroke outcomes following gut dysbiosis. We evaluated plasma SCFA concentrations against stroke severity parameters and identified SCFA-associated protein networks.

**Methods:**

The Blood and Clot Thrombectomy Registry and Collaboration (BACTRAC), a continuously enrolling tissue bank, was used to obtain stroke samples. Arterial blood distal and proximal to the thrombus was obtained from Acute Ischemic Stroke (AIS) Patients (n=53) during thrombectomy. Patient demographics, stroke presentation and outcome parameters were reported. The SCFAs were isolated from proximal plasma *via* chemical derivatization UHPLC coupled tandem mass spectrometry using electrospray ionization and multiple reaction monitoring. Proteomic levels for 184 cardioembolic and inflammatory proteins was quantified from systemic and intracranial plasma by Olink. Arterial blood from cerebrovascular patients undergoing elective neurointerventional procedures was used as controls.

**Results:**

Acetate positively correlated with time from last known normal (LKN) and was significantly lower in stroke patients compared to control. Isobutyrate, Butyrate and 2-Methylbutyrate negatively correlated with %ΔNIHSS. Isobutyrate and 2-Methylbutyrate positively correlated with NIHSS discharge. SCFA concentrations were not associated with NIHSS admission, infarct volume, or edema volume. Multiple SCFAs positively associated with systemic and pro-inflammatory cytokines, most notably IL-6, TNF-α, VCAM1, IL-17, and MCP-1.

**Conclusions:**

Plasma SCFA concentrations taken at time of stroke are not associated with stroke severity at presentation. However, higher levels of SCFAs at the time of stroke are associated with increased markers of inflammation, less recovery from admission to discharge, and worse symptom burden at discharge.

## Introduction

Acute ischemic stroke (AIS) remains a significant health burden in the United States. The localized brain damage following AIS includes peripheral signaling to immune cells to infiltrate the brain and impede and/or assist in mechanisms of recovery. As a result, the systemic circulation mounts an acute inflammatory state that may also cause dysfunction in other organs, such as lung, heart, and gut (1–5). In fact, the microbiome in the gut is disrupted (dysbiosis) following AIS, as well as in comorbidities associated with AIS (e.g., hypertension, metabolic changes, inflammatory changes, etc.) due to bi-directional communication of the gut and brain (gut-brain axis) (6–8). The microbiome is needed to maintain a healthy balanced gut. Following AIS, increased dysbiosis is associated with worse brain injury and poorer prognosis (9). Though the exact mechanism of the interplay between gut dysbiosis and stroke is still unknown, dysregulations in the gut-brain axis alters the subsequent immune response as well as other important signaling pathways, which ultimately may lead to impaired recovery and worse outcomes.

Short Chain Fatty Acids (SCFAs) are products of gut microbiome enabled bacterial fermentation of dietary fructans with important roles in systemic energy metabolism that also act as bioactive mediators for numerous biologic functions and organ systems, some of which are still being determined (4). The gut microbiome helps maintain immune, nutritional, and metabolic homeostasis and development, and SCFAs aid in these functions (4, 10). Peripherally, SCFAs help modulate intestinal lymphocytes and monocytes, while in the brain they enhance microglial activation and morphology (5, 11, 12). These SCFA effects are interlinked with changes in expression profiles of various cytokines essential for mediating inflammatory responses and immune cell maturation. Several described associations included promotion of the anti-inflammatory cytokine interleukin (IL)-10, decreased expression of the pro-inflammatory Vascular Cell Adhesion Molecule 1 (VCAM1), and suppression of the pro-inflammatory cytokines: Tumor Necrosis Factor (TNF)-α, Monocyte Chemoattractant Protein (MCP)-1, IL-17, IL-1β, and IL-6 (13–17). These actions are predominantly carried out through activation of G-protein coupled receptors and free fatty acid receptors, as well as inhibition of histone deacetylase (18, 19). These effects may significantly impact the immune response in AIS and is one driving force behind the increasing interest for research of SCFAs in AIS.

SCFA concentration profiles and gut microbiota integrity are altered in various neurodegenerative diseases including Alzheimer’s disease, Parkinson’s disease, amyotrophic lateral sclerosis, and multiple sclerosis (20–23). Studies have further investigated the effects that the SCFAs Acetate, Propionate, and Butyrate have in ischemic-related neurologic conditions. Butyrate specifically has shown efficacy in having neuroprotective properties (24, 25). SCFAs can reduce apoptosis and inflammation while promoting the maintenance of blood brain barrier (BBB) integrity following reperfusion (5, 9, 25). As the functions of SCFAs in stroke are becoming more apparent, studies have attempted using them as therapeutic agents in stroke models. The administration of SCFAs and SCFA-producing bacteria were efficacious in improving post-stroke neurological and motor functions, providing antioxidant and anti-apoptotic effects in stroke pathology, and protecting against BBB disruption and edema (5, 25–27). From these findings, it is no surprise that fecal and plasma SCFA concentration profiles are altered the days following AIS secondary to gut dysbiosis, and these changes are associated with worse outcomes (5, 28).

The current study utilized the Blood and Clot Thrombectomy registry and collaboration (BACTRAC) protocol (clinicaltrials.gov; NCT03153683) to obtain samples of arterial blood proximal (i.e. systemic) and distal (i.e. intracranial) to the thrombus in human patients during mechanical thrombectomy following AIS. This registry obtains high-quality specimens that provide a means to investigate the initial molecular response in AIS and compare those with patient demographics and stroke parameters (29). Previously published studies that used this protocol have provided novel data relating proteomic characteristics with clinical outcomes. These studies have shown that there are significantly lower intracranial expressions of proteins involved in th2 and neutrophil proliferation [i.e., C-C motif chemokine 19 (CCL19), 20 (CCL20), and 23 (CCL23)] during stroke (30) and that the expression of various cytokines, chemokines, and inflammatory proteins taken at the time of thrombectomy may predict stroke severity (30–33). More so, alterations in peri-infarct blood gases and electrolytes were found to exist during stroke, and some of these are sex-specific (34, 35).

In most of the published literature to date relating to AIS, SCFAs were primarily analyzed in animal models that looked at concentrations of fecal SCFAs and their bacterial source, as well as plasma SCFA concentrations the days following stroke. Human studies have examined fecal samples of SCFAs and their bacterial source from patient feces following first defecation after stroke, while one study looked at blood capillary levels of the ketone and SCFA, β-hydroxybutyrate, at the time of admission (28, 36, 37). We found no studies, human or animal, that evaluated the plasma levels of the SCFAs Acetate, Propionate, Isobutyrate, Butyrate, and 2-Methylbutyrate at the time of stroke. This study used patient’s plasma from systemic arteriolar blood taken during thrombectomy to quantify the levels of the these SCFAs and compare them with patient demographics, stroke presentation and discharge parameters, and plasma proteomic data. From these analyses, we aim to identify clinical and molecular associations of AIS-induced injury with the levels of circulating SCFAs at time of stroke onset.

## Methods

### Primary Sample Acquirement and Processing

BACTRAC is a continually enrolling tissue bank and registry that was used to obtain human plasma samples at the time of thrombectomy in AIS patients. The study is approved through the Institutional Review Board (IRB) at the University of Kentucky, and its banking and registry methods have been previously described (29). All non-pregnant, non-prisoner thrombectomy patients of adult age (≥18 y.o.) were considered, and written consent was obtained from the patient or their legally-appointed representative prior to enrollment. Subjects for whom consent could not be obtained within 72 hrs of thrombectomy were excluded. Patient demographics including age, sex, BMI, smoking status, hypertension (HTN), diabetes mellitus 2 (DMII), hyperlipidemia (HLD), previous stroke, and stroke severity parameters including Modified Rankin Scale (mRS) Premorbid and Discharge scores, National Institutes of Health Stoke Scores (NIHSS) on admission and discharge, thrombolysis in cerebral infarction (TICI) Score, computed tomography angiogram (CTA) collateral score, infarct and edema volumes, and time from last known normal (LKN) to thrombectomy (in minutes) were recorded.

From the stroke patient population, systemic (proximal to the thrombus) and intracranial (distal to the thrombus) blood samples were obtained and isolated for plasma. The tissue processing and protein isolation methods have been previously described (30). Olink (Olink Proteomics, Boston, MA) was given both intracranial and systemic plasma samples to analyze and quantify proteomic data for 184 cardiometabolic and inflammatory proteins. They were used because of their well-established methods pertaining to proteomic analyses on small-volume samples, which was of particular interest to BACTRAC since our intracranial sample volumes are small. Using proximity extension assay (PEA) technology, Olink reports a Normalized Protein eXpression (NPX) value that is a unit in log2 scale instead of providing protein expression as a concentration value, allowing for the analysis of individual protein across a given sample set.

Provided with patient-matched systemic plasma samples only, the University of Kentucky’s Small Molecule Mass Spectrometry Core Lab determined SCFA concentrations. The quantification methods were performed based on previously published protocols (38, 39), using chemical derivatization with ultra-performance liquid chromatography and tandem mass spectrometry (UPLC-MC) with stable isotope-labeled internal standards. SCFA concentrations were reported in micromoles (μM) that was calculated using the instrument response to the internal standards.

### Cerebrovascular Control Samples

A cohort of patients (n=12) with stroke risk factors who were undergoing minimally invasive diagnostic angiograms and elective neurointerventional procedures (non-acute stroke, eg. carotid stent placement) were enrolled as the study’s cerebrovascular disease (CVD) control group. Of note, these patients were not presenting with AIS. Pre-existing demographics like those recorded for stroke patients were obtained. During procedures, arterial blood samples were taken from these patients and plasma was isolated and processed in accordance with the BACTRAC protocol. Protein and SCFA data were obtained from Olink and the Small Molecule Mass Spectrometry Core Lab, respectively.

### Statistical Analyses

Patient demographics were categorized into nominal (i.e., sex, comorbidities), ordinal (i.e., smoking status, mRS, TICI) and quantitative (i.e., age, LKN, infarct volume) variables. Several variables demonstrated unique considerations and were grouped in manners beyond this. BMI and NIHSS scores were each also stratified into ordinal groups as depicted in **Table 1**. NIHSS was further evaluated by calculating the difference percent change (%ΔNIHSS) between the admission scores and discharge scores as follows:


$$\%\Delta NIHSS=\frac{NIHSS\;Admission\;Score-NIHSS\;Discharge\;Score}{NIHSS\;Admission\;Score}$$

**Table 1:** Demographics and troke sparameters of thrombectomy patients (n=53).

	Value (%)		Value (%)
**Age (Median; range)**	68 (25-96)	**NIHSS Admission Scores** *	
**Sex**		Mild Stroke (1-4)	1 (2)
Female	32 (60)	Moderate Stroke (5-14)	17 (32.5)
Male	21 (40)	Severe Stroke (15-24)	27 (52)
**BMI** *		Very Severe Stroke (>24)	7 (13.5)
<18.5	1 (2)	**NIHSS Discharge Score** ***	
18.5-24.9	16 (31)	Mild Stroke (1-4)	15 (37.5)
25-29.9	22 (42)	Moderate Stroke (5-14)	16 (40)
30-34.9	8 (15)	Severe Stroke (15-24)	9 (22.5)
35+	5 (10)	**CTA Collateral** ****	
**Comorbidities**		0	8 (22)
Hypertension	41 (77)	1	24 (67)
Diabetes Mellitus II	17 (32)	2	4 (11)
Hyperlipidemia	12 (23)	**mRS Premorbid** ***	
Previous Stroke	10 (19)	0	18 (45)
**Smoking Status** **		1	12 (30)
Current	12 (27)	2	6 (15)
Previous (>6 mon)	6 (13)	3	2 (7.5)
Never	27 (60)	4	1 (2.5)
**TICI Score at Presentation**		**mRS Discharge** *****	
1 = Perfusion Limited	1 (2)	0	3 (11.5)
2A <= 50% Perfusion	2 (4)	1	4 (15)
2B >= 50% Perfusion	22 (41)	2	2 (8)
3 = Full Perfusion	28 (53)	3	1 (4)
		4	9 (35)
		5	4 (15)
		6	3 (11.5)

Values are median with range, mean ± SD, or (%).

N=53 Patients.

* 1 patient’s data missing (n=52).

** 8 patient’s data missing (n=45).

*** 13 patient’s data missing (n=40).

**** 17 patient’s data missing (n=36).

***** 27 patient’s data missing (n=26).

The use of %ΔNIHSS in acute AIS has been previously identified as an effective means of predicting long-term outcomes in AIS patients (40). A positive association with %ΔNIHSS would therefore depict better symptom recovery between admission and discharge as a %ΔNIHSS=1 depicts full symptom recovery, while a negative association depicts less recovery where %ΔNIHSS=0 indicates no symptom improvement.

Parametric tests included two tailed t-tests, Pearson correlation analyses, and linear regression analyses. Nominal patient demographics were analyzed with the SCFAs using two-tailed t testing to identify whether a significant difference of SCFA concentrations exists based on the presence of specific demographics. The Levene’s test of equality was incorporated into t-testing to account for population variance. Ordinal variables including TICI scores, mRS scores, smoking status, and the stratified variables were analyzed with SCFAs using one way ANOVA to identify mean concentration differences between groups. When ANOVA findings were significant, contrast testing was performed to identify the specific inter-variable groups that were significantly different (i.e. TICI score of 1 vs TICI score of 2B). Homogeneity of variance testing was used to identify population variance, and Tamhane’s T2 analyses were used when homogeneity was not present. Continuous, quantitative patient data (i.e. infarct, age) were analyzed with Pearson correlations and linear regression analyses to find correlations between SCFAs and these variables. Significance was set at p=0.05 and had a maximum sensitivity of p<0.001.

The use of a large number of correlation analyses on multiple quantitative variables within the same population increases the likelihood of identifying false-positive correlations. The SCFA analyses with nominal and ordinal patient data utilized mean differences that accounted for population variance to control for this. The SCFA analyses that looked at associations with continuous stroke parameter variables incorporated linear regression analyses to decrease the likelihood of false-positive findings. We did not control for the increased likelihood of false associations in the initial SCFA-protein correlations as these were used to identify all proteins with a possible association with the SCFAs, and these results were therefore not overtly highlighted (**Supplementary Tables 1** **–** **3**). The subsequent analyses did however compensate for this by only using proteins with >3 associations as well as those with correlations to both SCFAs and patient presentation variables. Correlations were considered significance if p ≤ 0.05, while they were noted as trends if 0.05<p ≤ 0.06.

Our cohort quantified SCFA data from patient’s systemic blood, while proteomic data was recorded from intracranial and systemic blood. The difference in protein levels between the intracranial and systemic plasma was calculated:


[ΔProtein=Intracranial protein levels−systemic protein levels]

This was done to provide a means of evaluating the magnitude of change between the cerebrovascular environment and the systemic blood during acute AIS. SCFA concentrations were individually correlated using Pearson correlations with protein levels from intracranial plasma, systemic plasma, and their difference. The correlated proteins were then studied relative to stroke presentation parameters (NIHSS admission, Infarct Volume, Edema Volume) using Pearson correlation analyses and linear regression. Due to non-normal distribution, infarct and edema volumes were log2 transformed to reach normality. Proteins that demonstrated significant clinical correlations were grouped according to their respective SCFA correlation. Multi-linear regression analyses were attempted to model both proteins and SCFAs together to predict these clinical parameters as well as multiple SCFAs to predict a given protein. NIHSS discharge, %ΔNIHSS and ordinal stroke parameters (i.e. TICI, mRS) were not used in the protein analysis due to the discrepancy between the SCFA-protein analysis population size (n=53) and the protein-presentation parameter analysis population sizes (n<53).

To identify protein networks associated with SCFAs, STRING V11.5 (https://string-db.org/) was utilized. This genome-wide proteomic database identifies physical and functional associations between proteins using proteomic connectivity integrations and database text mining. Networking was performed on proteins that demonstrated multiple (>3) SCFA correlations based on their SCFA groups. Furthermore, networking was independently performed for all systemic and intracranial proteins that exhibited >3 SCFA correlations. Proteins that had significant correlations with both SCFAs and stroke presentation parameters were clustered based on their respective SCFA correlations and networked. Output biological processes from STRING were reported for all networks based on the processes with the most significant false discovery rates.

## Results

### Patient Demographics/Findings

Our patient population consisted of 53 adults who underwent thrombectomy. The median age was 68 (25-96) where 32 (60.4%) patients were female. Hypertension was present in 41 patients, making up a significant portion of our patient population (77.4%). Furthermore, 27 (50.9%) of the patients never smoked, 6 (11.3%) were previous smoker, and 12 (22.6%) were current smokers; 8 (15.1%) patients did not report smoking status. On admission, 1 (1.9%) patient had a mild stroke (NIHSS 1-4), 17 (32.1%) had a moderate stroke (NIHSS 5-14), 27 (50.9) had a severe stroke (NIHSS 15-24), and 7 (13.2%) had a very severe stroke (NIHSS>25) according to NIHSS scores. One (1.9%) patient’s NIHSS admission score was not obtained. At discharge, 15 (28.3%) patients still have mild stroke disabilities, 16 (30.2%) had moderate stroke disabilities, and 9 (17%) had severe stroke disabilities based on NIHSS scores. Patients were discharged on average 9.95±7.89 days after admission. Of note, discharge scores were not obtained for 13 (24.5%) patients. Patient demographics including comorbidities and stroke outcomes are shown in **Table 1**.

Differences in SCFA concentrations taken from patient’s systemic blood were evaluated relative to patient demographics and found no differences between SCFA concentrations based on sex. Age was found to be weakly correlated with Propionate (P=0.038; R=0.285), Isobutyrate (P=0.016; R=0.328), and 2-Methylbutyrate (P=0.023; R=0.312; **Table 2**). Increased concentrations of Acetate (p=0.01) and Isobutyrate (p=0.005) were significantly higher in patients with comorbid hypertension, and Propionate and 2-Methylbutyrate mean concentrations were higher in hypertensive patients, but these findings fell short of meeting statistical significance (p=0.068 and p=0.059, respectively; **Table 2**). No associations in SCFA concentrations were observed with smoking status, DMII, hypercholesterolemia or previous stroke.

**Table 2:** Differences in plasma SCFA concentrations between nominal patient demographics and comorbidities (top) and Pearson Correlations between quantitative patient demographics and SCFA concentrations (bottom).

Nominal Data			Acetate (μM)			Propionate (μM)			Isobutyrate (μM)			Butyrate (μM)			2-Methylbutyrate (μM)		
		N	Mean	SD	p value	Mean	SD	p value	Mean	SD	p value	Mean	SD	p value	Mean	SD	p value
**Sex**	Female	32	68.40	56.59	0.466	1.64	1.17	0.251	0.32	0.28	0.914	0.45	0.47	0.726	0.42	0.27	0.908
	Male	21	80.32	59.58		2.08	1.52		0.33	0.39		0.40	0.65		0.41	0.26	
**HTN**	Yes	41	79.79	63.43	0.01	2.00	1.34	0.068	0.37	0.36	0.005	0.44	0.60	0.867	0.45	0.28	0.059
	No	12	50.34	16.84		1.20	1.11		0.19	0.09		0.41	0.31		0.29	0.12	
**DMII**	Yes	17	86.28	61.17	0.257	2.20	1.54	0.145	0.36	0.42	0.653	0.34	0.56	0.394	0.49	0.36	0.222
	No	36	66.91	55.52		1.63	1.19		0.31	0.28		0.48	0.54		0.38	0.20	
**HLD**	Yes	12	93.91	86.17	0.318	1.76	1.00	0.877	0.31	0.23	0.796	0.48	0.64	0.74	0.46	0.28	0.501
	No	41	67.04	45.75		1.83	1.42		0.33	0.35		0.42	0.52		0.40	0.26	
**Previous Stroke**	Yes	10	67.42	47.52	0.731	2.10	1.32	0.453	0.45	0.42	0.198	0.53	0.70	0.528	0.53	0.29	0.115
	No	43	74.45	60.03		1.75	1.33		0.30	0.30		0.41	0.51		0.39	0.25	
**Quantitative Data**		**N**	**R Value**		**p value**	**R Value**		**p value**	**R Value**		**p value**	**R Value**		**p value**	**R Value**		**p value**
**Age**		53	0.197		0.158	.285*		0.038	.328*		0.016	0.223		0.108	.312*		0.023
**BMI**		52	-0.104		0.461	-0.061		0.67	-0.104		0.462	-0.097		0.496	-0.158		0.264
**LKN**		50	.316*		0.026	0.067		0.645	0.239		0.094	0.081		0.576	0.235		0.1

*p values ≤ 0.05.

### SCFA Level Changes Due to Stroke

**Figure 1:**
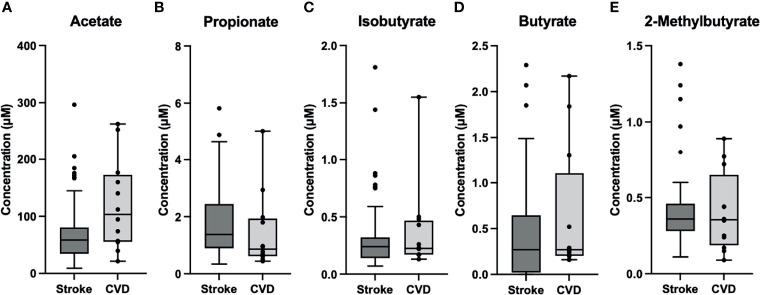
Box and Whisker Plots of Stroke (n=53) vs CVD Control (n=12). **(A)** Acetate was significantly lower (p=0.02) in stroke (M=73.1, SD=57.5) compared to control (M=120.6, SD=80.0). **(B–E)** Propionate (M_S_= 1.8, SD_S_=1.3; M_C_=1.44, SD_C_=1.35), Isobutyrate (M_S_=0.33, SD_S_=0.33; M_C_=0.38, SD_C_=0.39), Butyrate (M_S_=0.43, SD_S_=0.54; M_C_=0.63, SD_C_=0.72), and 2-Methylbutyrate (M_S_=0.41, SD_S_=0.26; M_C_=0.41, SD_C_=0.26) were comparable between groups. )M_S_= Stroke Mean, SD_S_= Stroke Std. Dev.; M_C_= Control Mean, SD_C_=Control Std. Dev.).

**Figure 2:**
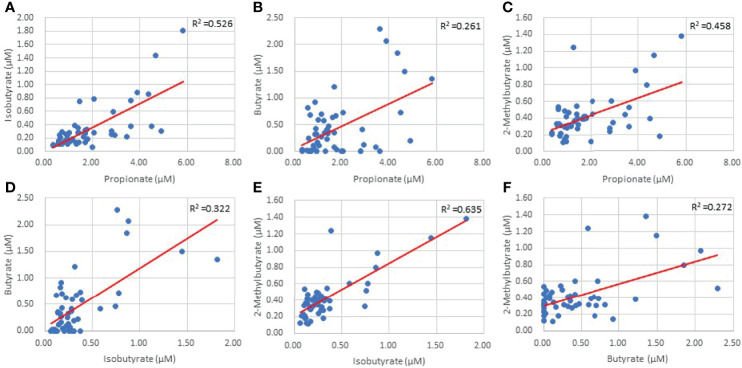
Inter-individual SCFA linear regressions of Propionate and Isobutyrate **(A)** Propionate and Butyrate **(B)** Propionate and 2-Methylbutyrate **(C)** Isobutyrate and Butyrate **(D)** Isobutyrate and 2-Methylbutyrate **(E)** and Butyrate and 2-Methylbutyrate **(F)** (p=0.00).

LKN was used as a means of measuring changes in SCFA concentrations as pathology progresses in the acute phase of stroke. Acetate was positively correlated with time since infarct and was the only SCFA that exhibited a significant correlation (p=0.026; R^2 ^= 0.10; **Table 2**). Differences between SCFA levels in CVD control and stroke patients were analyzed to identify potential changes in SCFAs due to stroke. Of note, CVD controls had a significantly lower mean age (mean= 50.27) than the stroke patients (mean= 66.89) (p<0.001). Interestingly, Acetate represented the only SCFA to be significantly different between stroke and CVD control patients with a mean concentration lower following AIS (p=0.02; t=-2.392; **Figure 1**), and this finding was preserved when accounting for outliers in stroke SCFA concentrations. Lastly, inter-individual SCFA concentration associations were analyzed. Propionate, isobutyrate, butyrate, and 2-methylbutyrate were all correlated with each other (p<0.001 for all; R^2^ = 0.272-0.635), while Acetate was not correlated with any of the other SCFAs (**Figure 2**).

### SCFA vs. Subacute Stroke Outcomes

**Figure 3:**
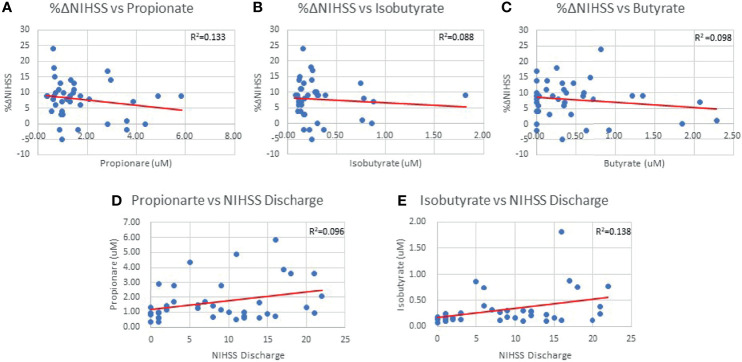
%ΔNIHSS correlations with Propionate (0.036) **(A)**, Isobutyrate (p=0.067) **(B)** and Butyrate (p=0.053) **(C)**. Propionate (p=0.051) **(D)** and Isobutyrate (p=0.018) **(E)** correlations with NIHSS Discharge scores.

Both infarct and edema volumes were studied relative to SCFA concentrations, but no correlations were found between any SCFAs and either infarct or edema volume (**Table 3**), and this finding was preserved when controlling for sex and hypertensive status. NIHSS score on admission was not correlated with SCFA concentrations (**Table 3**) and mean SCFA concentrations differences were not present when NIHSS admission score was stratified. The average duration of hospital stay from admission to discharge for the patient population was 10±8 days. Isobutyrate was positively correlated with NIHSS score at discharge (p=0.018; R^2^ = 0.138; **Table 3** and **Figure 3**), while only an association was demonstrated with Propionate (p=0.051; R^2^ = 0.096; **Table 3** and **Figure 3**). Mean concentrations of Propionate, Isobutyrate and Butyrate were higher in patients with NIHSS discharge scores >15 compared to those <5, though testing significance was limited due to non-homogenous variance across these SCFAs. Propionate was negatively correlated with %ΔNIHSS (p=0.036; R^2^ = 0.114; **Table 3** and **Figure 3**), while Isobutyrate and Butyrate exhibited trends for negative correlation (p=0.067; R=-0.296 and p=0.053; R=-0.313, respectively; **Table 3** and **Figure 3**). Acetate was the only SCFA that demonstrated significant variance between TICI scores (p=0.047), where patients with <50% perfusion (TICI=2A) had higher Acetate concentrations than those with full perfusion (TICI=3). Finally, 2-Methylbutyrate was higher in patients with moderate disability (mRS=3) compared to those with no significant disability (mRS=1) prior to stroke onset. No differences were found with CTA collateral score, mRS at discharge, or ΔmRS scores.

**Table 3:** SCFA pearson correlations with stroke parameters.

		Acetate	Propionate	Isobutyrate	Butyrate	2-Methylbutyrate
**Infarct Volume**	R value	-0.01	0.122	0.04	-0.071	-0.127
	p value	0.943	0.386	0.777	0.615	0.365
	N	53	53	53	53	53
**Edema Volume**	R value	0.086	0.095	0.036	-0.06	-0.007
	p value	0.545	0.501	0.801	0.672	0.959
	N	52	52	52	52	52
**NIHSS Admission Score**	R value	0.157	0.099	0.144	0.034	0.146
	p value	0.267	0.486	0.308	0.811	0.303
	N	52	52	52	52	52
**NIHSS Discharge Score**	R value	0.11	0.31	.371*	0.259	0.265
	p value	0.498	0.051	0.018	0.107	0.098
	N	40	40	40	40	40
**%ΔNIHSS**	R value	0.043	-.337*	-0.296	-0.313	-0.205
	p value	0.794	0.036	0.067	0.053	0.21
	N	39	39	39	39	39

No SCFAs were correlated with Infarct Volume, Edema Volume, or NIHSS Admission Score (p>0.05). Isobutyrate (p=0.018) was significantly correlated with NIHSS Discharge Scores. Propionate (p=0.036) was significantly correlated with %ΔNIHSS Scores. *p values ≤ 0.05.

### SCFA-Protein Correlations

A total of 87 systemic proteins (**Supplementary Table 1**), 87 intracranial proteins (**Supplementary Table 2**), and 53 Δproteins (**Supplementary Table 3**) significant correlations were found with SCFAs (p≤0.05). Isobutyrate and 2-Methylbutyrate exhibited the highest number of systemic and intracranial correlations among the SCFAs, though they both had the lowest number of Δprotein correlations. Butyrate upheld the majority of ΔProtein correlations. Acetate and Propionate had similarly distributed correlations across systemic, intracranial, and Δprotein groups.

**Figure 4:**
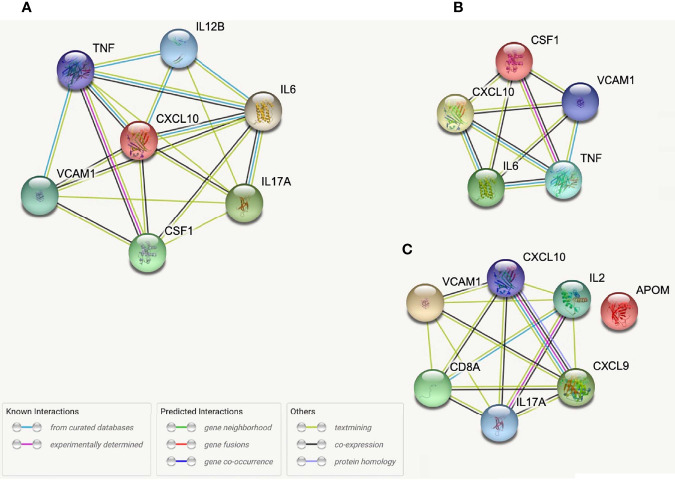
STRING protein networks for all intracranial proteins with >3 SCFA correlations **(A)**, intracranial proteins correlated with Propionate, Isobutyrate, Butyrate, and 2-Methylbutyrate **(B)** and all systemic proteins with >3 SCFA correlations **(C)**.

While no proteins demonstrated correlations across all five SCFAs when studied relative to sample location (i.e., systemic, intracranial), several proteins were correlated across multiple (4/5) SCFAs. Three systemic proteins (Apolipoprotein M (APOM), C-X-C motif chemokine ligand (CXCL)10, and IL-2 and six intracranial proteins (Colony stimulating factor-1 (CSF-1), CXCL10, IL-12B, IL-6, TNF-α, and VCAM1 (**Figure 4**) were correlated with Propionate, Isobutyrate, Butyrate, and 2-Methylbutyrate. Systemic CXCL9 and Cluster of Differentiation 8A (CD8A), as well as intracranial IL-17A, were correlated with Acetate, Isobutyrate, Butyrate, and 2-Methylbutyrate. Systemic VCAM1 and IL-17A correlated with Acetate, Propionate (p=0.053*), Isobutyrate, and 2-Methylbutyrate. Of these proteins, the only one that was significant by multi-linear regression with SCFAs was systemic IL-2 (R^2^ = 0.261), which interestingly had Acetate (p=0.009) and Isobutyrate (p<0.001) as predictors of IL-2 levels. STRING Networks were created separately using all systemic proteins with >3 SCFA correlations and all intracranial proteins with >3 SCFA correlations (**Figure 4**) and the associated biological processes were reported (**Table 4**).

**Table 4:** Biological processes associated with STRING networks for all intracranial proteins with >3 SCFA correlations (a), intracranial proteins correlated with propionate, isobutyrate, butyrate, and 2-methylbutyrate (b) and all systemic proteins with >3 SCFA correlations (c).

A. All Intracranial Proteins Correlated with >3 SCFAs			
Biological Process	Strength	FDR	Matching Proteins in Network
Cytokine-mediated signaling pathway	1.46	1.09E-06	CSF-1, CXC10, IL-12B, IL-17A, IL-6, TNF, VCAM1
Positive regulation of osteoclast differentiation	2.62	1.57E-06	CSF-1, IL-12B, IL-17A, TNF
Defense response to other organism	1.34	2.62E-06	CSF-1, CXC10, IL-12B, IL-17A, IL-6, TNF, VCAM1
Positive regulation of immune system process	1.31	2.84E-06	CSF-1, CXC10, IL-12B, IL-17A, IL-6, TNF, VCAM1
Cell activation	1.26	4.52E-06	CSF-1, CXC10, IL-12B, IL-17A, IL-6, TNF, VCAM1
Positive regulation of response to external stimulus	1.52	4.94E-06	CSF-1, CXC10, IL-12B, IL-17A, IL-6, TNF
Leukocyte migration	1.65	2.57E-05	CXC:10, IL-17A, IL-6, TNF, VCAM1
Immune response	1.09	2.57E-05	CSF-1, CXC:10, IL-12B, IL-17A, IL-6, TNF, VCAM1
Leukocyte differentiation	1.62	3.02E-05	CSF1, IL6, IL17A, TNF, VCAM1
Positive regulation of cytokine production involved in inflammatory response	2.6	4.79E-05	IL-17A. IL-6, TNF
**B. Intracranial Correlations with Propionate, Isobutyrate, Butyrate, and 2-Methylbutyrate**			
**Biological Process**	**Strength**	**FDR**	**Matching Proteins in Network**
Positive regulation of leukocyte migration	2.04	0.00027	CSF-1, CXCL10, IL-6, TNF
Cytokine-mediated signaling pathway	1.46	0.00045	CSF-1, CXCL10, IL-6, TNF, VCAM1
Regulation of mononuclear cell migration	2.35	0.00092	CSF-1, CXCL10, TNF
Positive regulation of gliogenesis	2.21	0.00092	CSF-1, IL-6, TNF
Leukocyte migration	1.69	0.00092	CXCL10, IL-6, TNF, VCAM1
Leukocyte differentiation	1.67	0.00092	CSF-1, IL-6, TNF, VCAM1
Defense response to other organism	1.34	0.00092	CSF-1, CXCL10, IL-6, TNF, VCAM1
Positive regulation of cell population proliferation	1.33	0.00092	CSF-1, CXCL10, IL-6, TNF, VCAM1
Positive regulation of immune system cells	1.31	0.00092	CSF-1, CXCL10, IL-6, TNF, VCAM1
Cell activation	1.26	0.00092	CSF-1, CXCL10, IL-6, TNF, VCAM1
**C. All Systemic Proteins Correlated with >3 SCFAs**			
**Biological Process**	**Strength**	**FDR**	**Matching Proteins in Network**
Granulocyte migration	1.96	0.0177	IL-17A, CXCL9, CXCL10
Leukocyte migration	1.55	0.0177	IL-17A, CXCL9, CXCL10, VCAM1
Cytokine-mediated signaling pathway	1.31	0.0177	IL-17A, IL-2, CXCL9, CXCL10, VCAM1
Immune response	1.02	0.0177	CD8A. IL-17A, IL-2, CXCL9, CXCL10, VCAM1
Cell activation	1.11	0.0261	CD8A. IL-17A, IL-2, CXCL10, VCAM1
Cell surface receptor signaling pathway	0.86	0.0347	CD8A. IL-17A, IL-2, CXCL9, CXCL10, VCAM1
Regulation of myoblast fusion	2.41	0.048	CXCL9, CXCL10

FDR, False Discovery Rate.

**Figure 5:**
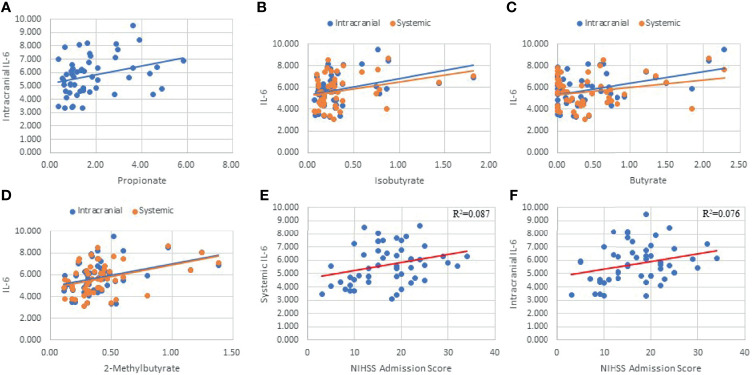
Linear Regressions of IL-6 with SCFAs and NIHSS admission score. Intracranial IL-6 was correlated with Propionate **(A)**, Isobutyrate **(B)**, Butyrate **(C)**, 2-Methylbutyrate **(D)**, and NIHSS Admission **(F)**. Systemic IL-6 was correlated with Isobutyrate **(B)**, Butyrate **(C)**, 2-Methylbutyrate **(D)**, and NIHSS Admission **(E)**.

Interleukin-6 (IL-6) taken from intracranial plasma demonstrated four SCFA correlations (Propionate: p=.026, R=0.305; Isobutyrate: p=0.01, R=0.353; Butyrate: p=0.002, R=0.407; 2-Methylbutyrate: p=0.004, R=0.385; **Figure 5** **)** as well as having systemic correlations with Isobutyrate (p=0.032; R=0.295) and 2-Methylbutyrate (p=0.003; R=0.398; **Figure 5**). Butyrate exhibited a trend correlation with systemic IL-6 (p=0.061; R=0.259; **Figure 5**) and was the only SCFA correlated with the ΔIL6 (p=0.037; R=0.288). IL-6 demonstrated a significant clinical profile in our study, including systemic (p=0.033; R^2 =^ 0.087; **Figure 5**) and intracranial (p=0.047; R^2 =^ 0.076; **Figure 5**) correlations with NIHSS admission. The mean systemic and intracranial levels of IL-6 were also significantly different between stratified NIHSS admission groups (p=0.032). On contrast testing, it was found that systemic (p=0.061) and intracranial (p=0.049) IL-6 were specifically higher in severe stroke (NIHSS = 15-24) compared to mild stroke (NIHSS < 5). Similar differences were found between very severe stroke and mild stroke, though these fell short of statistical significance. On linear regression, 2-Methylbutyrate was the most predictive SCFA for systemic IL-6 levels, while Butyrate was the most predictive of intracranial IL-6 levels, and the strength of both models improved when hypertension was included as a predictor in the models.

### Correlated Proteins vs. Stroke Severity at Presentation

Two systemic proteins (IL-20Rα: p=0.013, R=0.343 and Protein C Inactivator of Coagulation Factors Va and VIIIa (PROC): p=0.039, R=-0.288; **Table 5**) and one ΔProtein (IL-10Ra: p=0.039, R=-0.287) were correlated with Edema Volume, while no intracranial proteins were correlated. Systemic IL-20Ra was correlated with Propionate, Isobutyrate, and 2-Methylbutyrate, and PROC was correlated with Isobutyrate and Butyrate. Two ΔProteins (IL-10Rα: R=-0.322, p=0.019 and IL-13: p=0.019, R=-0.294) and one systemic protein (Regenerating islet-derived (REG)1A: p=0.055, R=-0.265; **Table 5**) were correlated with Infarct Volume, while no intracranial proteins demonstrated correlations. Butyrate was the only SCFA correlated with ΔIL-13, while no SCFAs were significantly correlated with ΔIL-10Rα (p=0.081). Systemic REG1A was correlated with Acetate, Isobutyrate, and 2-Methylbutyrate. We did not find any multi-linear regression models using both SCFAs and proteins to be predictive of infarct or edema volumes.

**Table 5:** Systemic proteins correlated with both SCFAs and stroke presentation parameters.

		Acetate	Propionate	Isobutyrate	Butyrate	2-Methylbutyrate
NIHSS Admission	(p=0.035) R=.293*)	**CA3** (p=0.049 R=.272*)				**CA3** (p=0.041 R=.282*)
	(p=<.001) R=.469**)	**CCL20** (p=0.018 R=.324*)		**CCL20** (p=0.008 R=.362**)		**CCL20** (p=0.001 R=.432**)
	(p=0.042) R=.284*)		**CCL4** (p=0.049 R=.272*)	**CCL4** (p=0.003 R=.407**)		**CCL4** (p=0.001 R=.437**)
	(p=0.05) R=0.273)			**CDCP1** (p=0.007 R=.364**)		
	(p=0.053) (R=0.27)				**IL-10** (p=0.029 R=.300*)	**IL-10** (p=0.038 R=.286*)
	(p=0.033) R=.296*)			**IL-6** (p=0.032 R=.295*)	**IL-6** (p=0.061 R=0.259)	**IL-6** (p=0.003 R=.398**)
	(p=0.048) R=.276*)			**LAP TGF-beta-1** (p=0.048 R=.273*)		**LAP TGF-beta-1** (p=0.03 R=.299*)
	(p=0.051) R=0.272)		**MCP-1** (p=0.031 R=.297*)			**MCP-1** (p=0.008 R=.359**)
	(p=0.036) R=.291*)					**NID1** (p=0.054 R=0.266)
	(p=0.044) R=.280*)			**OPG** (p=0.003 R=.398**)	**OPG** (p=0.001 R=.429**)	**OPG** (p=0.019 R=.321*)
	(p=0.041) R=.285*)					**SOD1** (p=0.002 R=.421**)
Edema Volume	(p=0.013) R=.343*)		**IL-20Ra** (p=0.034 R=.292*)	**IL-20Ra** (p=0.009 R=.356**)		**IL-20Ra** (p=0.001 R=.438**)
	(p=0.039) (R=-.288*)			**PROC** (p=0.012 R=-.342*)	**PROC** (p=0.028 R=-.302*)	
Infarct Volume	(p=0.055) (R=-0.265)	**REG1A** (p=0.02 R=.319*)		**REG1A** (p=0.033 R=.293*)		**REG1A** (p=<.001 R=.528**)

The p and R values listed in the second column are those for the stroke parameter-protein correlations. The p and R values listed under each SCFA column beside each protein are those for the SCFA-protein correlations.

*p values, 0.01-0.05. **p values ≤ 0.01.

**Figure 6:**
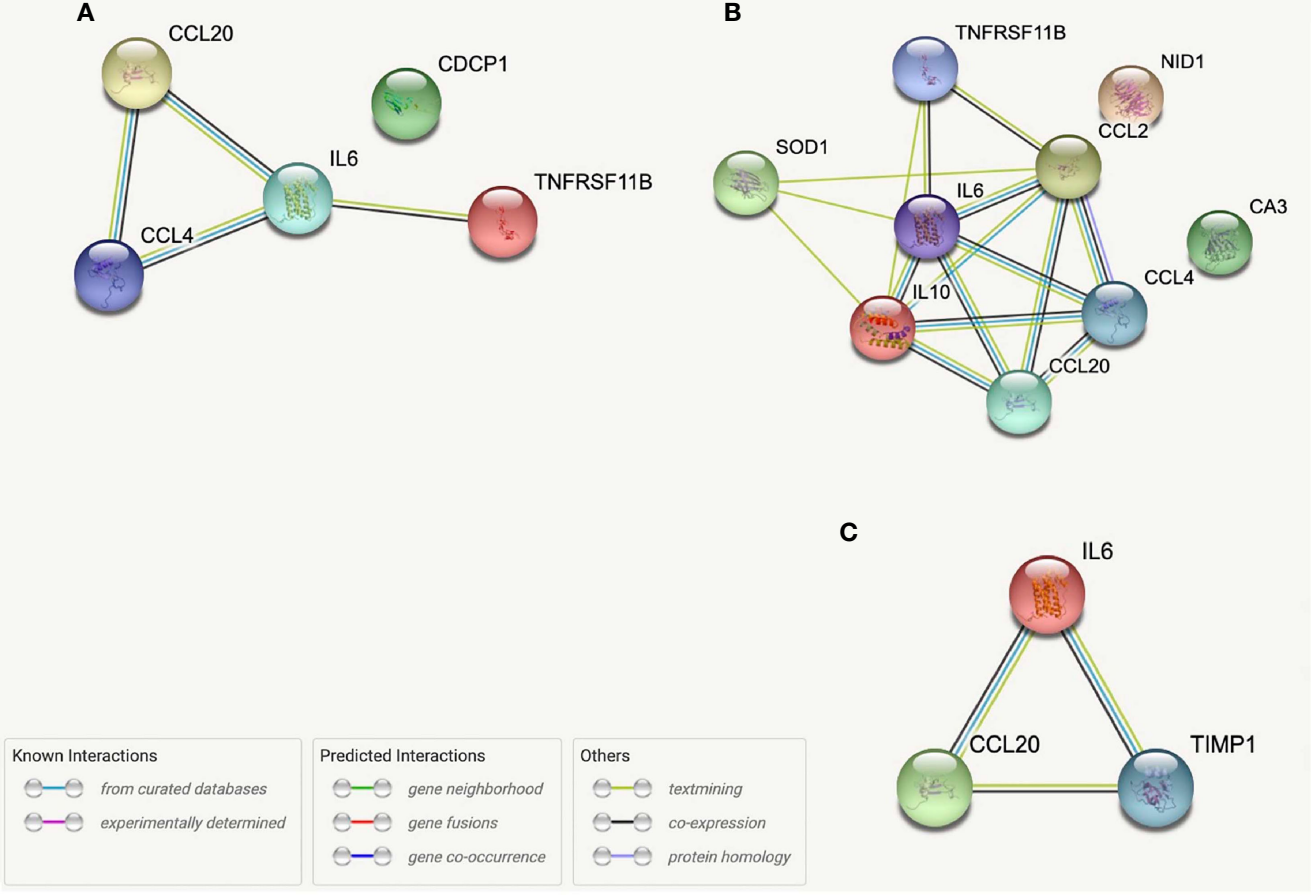
STRING protein networks for systemic proteins correlated with both Isobutyrate and NIHSS admission scores **(A)**, systemic proteins correlated with both 2-Methybutyrate and NIHSS admission scores **(B)** and intracranial proteins correlated with Isobutyrate, 2-Metlybutyrate and NIHSS Admission Scores **(C)**. *OPG, TNFRSF11B; MCP-1, CCL2; LAP-TGFB1 was not found in STRING.

From our entire proteomic data, ten systemic proteins, seven intracranial proteins and two ΔProteins were significantly (p ≤ 0.05) correlated with NIHSS admission; of these, seven systemic (**Table 5**) and two distal (**Table 6**) proteins also had SCFA correlations. Systemic IL-10 and MCP-1 demonstrated trending (0.05<p<0.06) correlations with NIHSS admission and were both correlated with 2-Methylbutyrate while IL-10 alone was also correlated with Butyrate. Nidogen (NID)-1 was one of the ten systemic proteins that were significantly correlated with NIHSS admission while it only upheld a trend correlation (p=0.054) again with 2-Methylbutyrate. One intracranial protein, Tissue inhibitor matrix metalloproteinase 1 (TIMP1), demonstrated multiple SCFA correlations but fell short of having statistical correlation with NIHSS admission (p=0.057). From these findings, Isobutyrate and 2-Methylbutyrate each supported a STRING network model with their respective systemic protein-clinical outcome correlations, while one STRING network model was used for both Isobutyrate and 2-Methylbutyrate together using intracranial proteins since they shared the same protein correlations (**Figure 6**). The most significant biological processes of these models were reported (**Table 7**).

**Table 6:** Intracranial proteins correlated with both SCFAs and NIHSS admission scores.

	Acetate	Propionate	Isobutyrate	Butyrate	2-Methylbutyrate
CCL20	(p=0.05) R=0.27)		(p=0.012) R=.344*)		(p=0.002) R=.412**)
	(p=0.001) R=.431**)		(p=0.001) R=.431**)		(p=0.001) R=.431**)
IL-6		(p=0.026) R=.305*)	(p=0.01) R=.353**)	(p=0.002) R=.407**)	(p=0.004) R=.385**)
		(p=0.047) R=.276*)	(p=0.047) R=.276*)	(p=0.047) R=.276*)	(p=0.047) R=.276*)
TIMP1			(p=<.001) R=.445**)	(p=0.022) R=.315*)	(p=0.001) R=.435**)
			(p=0.057) R=0.266)	(p=0.057) R=0.266)	(p=0.057) R=0.266)

SCFA-Protein p and R values are listed on the top of cell. Protein-NIHSS admission score p and R values are listed on the bottom of each cell. *p values = 0.01-0.05. **p values ≤ 0.01.

**Table 7:** Biological processes associated with STRING networks for systemic proteins correlated with both Isobutyrate and NIHSS admission scores (a), systemic proteins correlated with both 2-methylbutyrate and NIHSS admission scores (b) and intracranial proteins correlated with Isobutyrate, 2-methylbutyrate and NIHSS admission scores (c).

A. Systemic Proteins Correlated with Isobutyrate and NIHSS Admission Score			
Biological Process	Strength	FDR	Matching Proteins in Network
Monocyte chemotaxis	2.44	0.0021	CCL20, CCL4, IL-6
Positive regulation of leukocyte migration	1.91	0.0172	CCL20, CCL4, IL-6
Negative regulation of bone resorption	2.75	0.0183	IL-6, TNFRSF11B
Cytokine-mediated signaling pathway	1.36	0.0183	CCL20, CCL4, IL-6, TNFRSF11B
Cellular response to tumor necrosis factor	1.68	0.0288	CCL20, CCL4, TNFRSF11B
Positive regulation of lymphocyte migration	2.31	0.0474	CCL20, CCL4
**B. Systemic Proteins Correlated with 2-Methylbutyrate and NIHSS Admission Score**			
**Biological Process**	**Strength**	**FDR**	**Matching Proteins in Network**
Monocyte chemotaxis	2.31	3.70E-05	CCL2, CCL20, CCL4, IL-6
Leukocyte chemotaxis	1.88	3.70E-05	CCL2, CCL20, CCL4, IL-10, IL-6
Cytokine-mediated signaling pathway	1.35	3.70E-05	CCL2, CCL20, CCL4, IL-10, IL-6, SOD1, TNFRSF11B
Cell surface receptor signaling pathway	0.87	0.00057	CCL2, CCL20, CCL4, IL-10, IL-6, NID1, SOD1, TNFRSF11B
Lymphocyte chemotaxis	2.12	0.0025	CCL2, CCL20, CCL4
Regulation of leukocyte migration	1.62	0.0025	CCL2, CCL20, CCL4, IL-6
Positive regulation of MAPK cascade	1.3	0.0026	CCL2, CCL20, CCL4, IL-6, SOD1
Response to organic substance	0.76	0.0027	CA3, CCL2, CCL20, CCL4, IL-10, IL-6, SOD1, TNFRSF11B
Response to carbon monoxide	3.04	0.0029	IL-10, SOD1
Cellular response to tumor necrosis factor	1.55	0.003	CCL2, CCL20, CCL4, TNFRSF11B
**C. Intracranial Proteins Correlated with Isobutyrate, 2-Methylbutyrate and NIHSS Admission Score**			
**Biological Process**	**Strength**	**FDR**	**Matching Proteins in Network**
Cytokine activity	1.92	0.009	CCL20, IL-6, TIMP1

FDR, False Discovery Rate.

## Discussion

Gut dysbiosis begins in the hyperacute phase (within minutes to hours) following stroke and leads to increased gut permeability with cellular leakage from the gut into the blood (41). Studies have found that intestinal immune homeostasis is altered following stroke with immune cell (i.e. T Cell) translocation from the gut to the brain that impacts ischemic injury (42). SCFAs, most notably Butyrate, may be involved in these processes as they help maintain gut permeability and affect intestinal immune regulation (43). These bioactive mediators may translocate from the intestine into systemic circulation (11, 44) where they perform various functions that may impact stroke pathophysiology including peripheral immune regulation and freely crossing the BBB to act directly in the brain (45–47). However, the full understanding of and clinical implications for what processes occur with SCFAs immediately following stroke is underdetermined. Being the first study to look at systemic plasma SCFA concentrations in humans at the time of thrombectomy, we hypothesized that the SCFAs translocate into the blood early following stroke where they may modulate the peripheral immune response directly and the intracranial immune response indirectly through molecular pathways, which may impact stroke burden at presentation.

The gut microbiota is affected by differences among inter-individual patient demographics. Sex has been shown to help shape the gut microbiota (48), which would ultimately impact intestinal SCFA concentrations and potentially circulating concentrations. In a study that looked at the effects of sex differences in MCAO rats on gut permeability, males had higher Propionate, Butyrate, and Isobutyrate fecal levels at baseline 2 days prior to stroke than females (41); Acetate and 2-Methylbutyrate were not examined. In our study, sex did not have an identifiable effect on SCFA concentrations. Age has been associated with decreased gut microbiome diversity and lower fecal SCFA concentrations (49). Spychala, et. Al. found that aged gut flora, independent of the host’s age, was associated with lower fecal Acetate, Propionate, and Butyrate concentrations at baseline and a more pronounced decrease the days-weeks following MCAO stroke in mice (50). Though we did not analyze fecal content, our data supported a weak but positive correlation between age and plasma Propionate, Isobutyrate, and 2-Methylbutyrate concentrations.

Several of the comorbidities looked at in this study have been identified as risk factors for stroke, including hypertension, diabetes, obesity, dyslipidemia, smoking, and previous stroke (51). Decreased SCFA concentrations have been associated with several of these comorbidities, including hypertension, DM2, and obesity (52, 53). In a human study, however, higher risk stroke patients were found to have lower Butyrate-producing bacteria and lower fecal butyrate concentrations, while fecal concentrations of Acetate, Isobutyrate, and Propionate were comparable among all groups (54). Hypertension was present in an overwhelming portion of our population (77.4%), whereas other comorbidities were much less represented. We found that mean SCFA concentrations (except for Butyrate) were higher in patients with comorbid hypertension at the time of stroke, while no significant differences were found in DM2, obesity, dyslipidemia, or previous stroke at the time of stroke. More so, SCFA concentrations in CVD controls were comparable between all demographics. Therefore, we believe these findings may be a result of transient plasma SCFA concentrations due to the abrupt changes seen immediately following stroke, though higher plasma SCFA concentrations could also be present in the stroke population at baseline.

Despite considerable research and multiple studies, the exact processes driving changes in blood and fecal SCFA levels after AIS is still underdetermined with results varying between studies. Acetate, Propionate, and Butyrate when analyzed together were found to have decreased plasma total concentrations three days following stroke and lower fecal concentrations compared to control (5, 28). However, Propionate, Butyrate, and Isobutyrate fecal levels were not significantly different 2 days prior to MCAO occlusion compared to 2 days following occlusion in rats (41). We therefore aimed to analyze the differences between stroke and control to look for potential concentration changes and identify SCFA correlations with LKN to evaluate whether SCFA levels transiently correlate with pathology progression. Acetate was the only SCFA that demonstrated significant concentration differences following stroke based on our control analysis, and it was the only SCFA significantly correlated with LKN, indicating that Acetate levels positively associate with the time following infarct. The other SCFAs displayed a general positive association with LKN, but these were not significant. These findings have several interpretations, which requires consideration of model limitations regarding the size of our control population relative to stroke. First, Acetate may have an early decrease at the time of infarct with a subsequent increase as stroke pathology progresses, while other SCFAs do not significantly change immediately following stroke. Alternatively, these results may depict a gradual increase in all the SCFAs that is most pronounced in Acetate, while our control cohort either has uniquely high Acetate values or does not fully represent the general population due to small population size. The relative timeframe between infarct and sample obtainment as well as the use of human patients instead of animal subjects are likely contributions to the difference between our findings and those of other studies as well. Lastly, we cannot disregard the possibility that the duration of time from infarct to sample obtainment may be too short to observe significant changes in SCFA concentrations. Since this study provides a “snapshot” in time of molecular changes involved in acute stroke pathology, a definitive conclusion regarding the nature and extent of change compared to baseline cannot be drawn. Quantification of both plasma and gut SCFA levels prior to, at the time of, and regularly following AIS in a single study using the same subjects would be ideal and better illustrate the characteristics of these changes, though this is not possible to perform in human studies due to ethical dilemmas.

As previously mentioned, SCFAs have been associated with stroke severity and outcomes. Lower fecal Acetate and Propionate but not Butyrate concentrations or the concentrations of SCFA-producing bacteria taken within 72 hours after stroke were associated with worse 90 day post-stroke functional outcome (28), while higher concentrations have been shown to potentially improve outcomes observed days to months after the ischemic insult (5, 28). Sadler, et al. demonstrated that 4-week pretreatment of mice with Acetate, Propionate, and Butyrate improved motor deficits at 56 days post-stroke (5). Additionally, intranasal treatment with supraphysiologic levels of Sodium Butyrate 1hr following MCAO reduced infarct size and significantly improved neurologic function at 24 and 72 hours (25). Our study examined SCFA correlations with stroke severity at presentation and discharge as well as the change in severity from admission to discharge. Interestingly, the only clinical finding at presentation that related to SCFAs was that of Acetate being higher in patients with >50% perfusion compared to those with full perfusion, though this finding is limited due to having only two patients exhibit >50% perfusion in our cohort. We therefore conclude that plasma SCFAs taken during thrombectomy are not directly associated with stroke severity at presentation. Propionate was negatively correlated with %ΔNIHSS, while both Propionate and Isobutyrate were positively correlated with NIHSS discharge scores. These SCFAs were also found to have significantly higher plasma concentrations in patients who still had severe stroke functional deficits compared to patients with mild stroke functional deficits when looking at stratified NIHSS discharge scores. These results demonstrate that higher Propionate and Isobutyrate concentrations at thrombectomy are associated with worse patient stroke burden days following stroke and that higher Propionate alone may associate with reduced improvement from presentation to discharge. Since no associations were found for these SCFAs with other discharge or change parameters such as mRS scores, further analysis should be performed to confirm these findings.

One objective of our study was to explore the relationships between SCFAs and proteomic signaling networks that may be involved in the pathophysiology of AIS. Almost immediately after the initial insult in AIS, a strong inflammatory cascade is activated with translocation of P-selectin to the surface of vascular endothelium and platelets (55). This is subsequently accompanied by increased transcription of other molecules involved in leukocyte adhesion including E-selectin, VCAM1 and intercellular adhesion molecule 1 (ICAM1) (56) as well as progressive disruption of BBB integrity and permeability from oxidative stress and the effects of inflammatory mediators (57). The brain parenchyma concomitantly experiences its own inflammatory process with release of damage associated molecular patterns (DAMPs) from injured cells and activation of various immune cells such as astroglia and microglia that produce pro-inflammatory cytokines that have been associated with stroke severity, including IL-1β, IL-6 and TNFα (58, 59). More so, this intra-parenchymal immune response has been shown to increase the expression of chemoattractants for monocytes and T cells (i.e., CXCL10, MCP-1) as well as Tregs (i.e., CCL20) among others (60–63). Previous studies have found that the SCFAs Acetate, Propionate and Butyrate are able to decrease the secretion of TNFα and MCP-1 from cultured monocytes as well as TNFα-induced VCAM1 expression in endothelial cells (15, 17). The intracranial proteins that were most correlative with SCFAs in our study included CSF1, CXCL10, IL-12B, IL-17A, IL-6, TNFα and VCAM1, while CCL20, IL-6 and TIMP1 were those intracranial proteins also associated with NIHSS admission scores. Of these, the positive correlations with IL-6, TNFα, VCAM1, and CXCL10 would suggest indirect associations between the systemic SCFAs and these intracranial molecules that promote neuroinflammation through T cell, B cell, and microglial activation and migration. Correlating with CCL20 would promote Treg infiltration into the brain and depict the previously described association between SCFAs and decreased activity of various immune cells. The current study model cannot determine the exact relationship (i.e., inhibitory vs. stimulating) or nature (result of stroke vs contributing to stroke) between SCFAs and these proteins, but these findings do draw attention to pertinent intracranial processes previously described in stroke which may be influenced by SCFAs.

The leakage of DAMPs and cytokines through the BBB and CSF to systemic circulation, along with other systemic processes such as gut dysbiosis, help drive the peripheral immune response following AIS and contribute to the late immunosuppression characterized by prolonged lymphopenia and increased peripheral Treg cells (64, 65). CD4(+), CD8(+), regulatory (Treg) and δγ T cells as well as NK cells, neutrophils and B cells are among the different immune cells that migrate to the local brain environment following the initial inflammatory cascade where they provide both deleterious and protective effects by mediating inflammation and cellular repair (66–68). δγ T cells and Treg cells are of particular interest to the current study since they primarily reside in the intestines and have been shown to release into the systemic circulation due to stroke-induced gut dysbiosis (42). δγ T cells then migrate to the brain where they reside in the leptomeninges (but have been found in autoptic brain tissue after stroke) and secrete IL-17A, a cytokine found to be increased in stroke patients which aids in neutrophil invasion into the brain, increases chemokine secretion from other cells and promotes neuroinflammation (42, 69, 70). Anti-inflammatory IL-10 secreting Tregs can modulate the brain’s immune response indirectly from the periphery [i.e., by IL-10 inhibiting δγ T cells (42)] or infiltrate the brain through the chemokines CCL1 and CCL20 where it acts directly by inhibiting various immune processes, including astrogliosis (63). Systemically, the proteins that held multiple SCFA correlations in the present study include APOM, CCL20, CCL4, CXCL9 & 10, IL-10, IL-2, IL-6, IL-8, IL-17a, IL-20RA CD8A and VCAM1 while CCL20, CCL4, IL-6, IL-20RA, IL-10, Reg1A and osteoprotegerin (OPG) were among the prominent systemic proteins with both SCFA and clinical correlations. Of these, the only negative correlation was seen with Reg1A. These correlations depict potential involvement of the SCFAs in various immune processes, including Treg and δγ T cell migration and activity (IL-6, IL-10, IL-17A, CCL20) leukocyte adhesion and migration (CCL4, CXCL9/10, VCAM1) and modulation of the activity of CD8(+) T cells (IL-2, CD8A), CD4(+) T cells (IL-10, IL-6), NK Cells (IL-2), neutrophils (IL-8) Macrophages (IL-10, IL-6) and B Cells (IL-10, IL-20RA) (71). As previously mentioned, the underlining characteristics of these associations cannot be determined from the current study model. However, these findings highlight the peripheral cellular processes involved in stroke that SCFAs may potentially influence. Future studies can use the associations described here to guide their analyses of SCFA immunomodulatory mechanisms in different *in vitro* and *ex vivo* AIS models.

IL-6 is a pro-inflammatory cytokine that is involved in acute systemic inflammatory responses as well as delayed responses through activation of T and B cells that is of specific interest to this study since it has been associated with worse short-term stroke outcomes (72, 73). Higher plasma concentrations of IL-6 on the first day of stroke impacts the neurologic and functional status of patients early in disease progression (74). We found that higher systemic and intracranial levels of IL-6 were associated with higher NIHSS admission scores thereby further confirming previous studies’ results of IL-6’s role in the acute phase of stroke. Acetate, Propionate, and Butyrate have been shown to express inhibitory effects on LPS-induced expression of IL-6 in endothelial cells (17). In our study, both intracranial and systemic IL-6 demonstrated positive correlations with all SCFAs except Acetate. IL-6 was also present in all but one STRING protein network reported. The consistency of these correlations strongly supports an immunologic relationship between SCFAs and this cytokine. We strongly suggest that other studies further explore this potential relationship as it may provide valuable insight into the pathophysiology and risk factors for AIS as well as other pathologies that involve IL-6.

STRING allowed us to identify protein networks involved with these proteins that had significant associations with multiple SCFAs. We created a total of six models which consist of three that depict proteins with the most SCFA correlations and three which depict the proteins that were correlated with Isobutyrate and 2-Methylbutyrate as well as NIHSS admission scores. The most prevalent biological processes across all the networks were “Cytokine-mediated cell signaling” and “Leukocyte migration”. The outputs from each of the systemic models displayed a preference on processes that pertain to immune cell chemotaxis and migration, while the three intracranial models had a wider variance of outputs that included cytokine activity and signaling as well as leukocyte migration, among others. The biological processes “regulation of mononuclear cell migration” and “positive regulation of gliogenesis” from the Propionate, Isobutyrate, Butyrate, and 2-Methylbutyrate’s intracranial model supports previous findings that demonstrate SCFAs, specifically Butyrate, regulate microglia maturation and may affect the brain’s expression of CSF-1 (11), a macrophage differentiating cytokine that was present in the model (**Figure 4**). These results support the possible role of systemic SCFAs indirectly affecting the local inflammatory process in the brain by regulating both peripheral and intracranial cell signaling as well as leukocyte migration to the affected area. It also further suggests the potential involvement of SCFAs in the immunologic processes of specific immune cells involved in stroke that were described earlier. These findings provide insight into the biological functions that are possibly being affected by plasma SCFAs both indirectly and directly in stroke.

The results of this paper suggest that early increases in plasma SCFA concentrations at the time of stroke are associated with worse disability at discharge and higher levels of inflammatory markers that drive stroke pathology. Furthermore, our findings do not support an association between plasma SCFAs and stroke severity at presentation. We theorize three possible underlining reasons for these observations. First, higher levels of plasma SCFAs at this time may be a pathologic result of acute gut dysbiosis with displacement of SCFAs from the gut intestine to the plasma. Secondly, the shift of SCFAs from the gut to the systemic circulation could be protective where their release is the body’s attempt to provide negative feedback on the exacerbated immune response following AIS. Either of these interpretations are supported by the correlations we found with SCFAs and pro-inflammatory cytokines, though the associations with worse stroke severity better supports the former. Alternatively, the third possibility is that obtaining SCFA concentrations in the plasma at the time of thrombectomy may be too early in pathology to completely depict their role in gut dysbiosis and stroke, which in part is supported by the findings with LKN and CVD controls. We also identified proteins involved in different immune processes that were correlated with the SCFAs in this study, including those that influence the activity and migration of Tregs, δγ T cells, CD4(+) and CD8(+) T cells, B cells, NK Cells, Neutrophils, and microglia. Though we cannot draw conclusions from these SCFA-protein correlations, they provide novel insight into potential mechanisms of the role of SCFAs in stroke through immune modulation.

One limitation that may impact the results of this study was incomplete patient reports. Missing information on outcome parameters such as NIHSS discharge and mRS scores may impact the sensitivity of significance values in correlation analyses thereby creating false correlations or shadowing real ones. Our study included 53 stroke patients and 12 CVD controls who may have potential confounding variables and inherent heterogenicity that could limit conclusions. Moreover, this significant difference in population size between stroke and CVD negatively impacts the statistical power of their comparison and limits conclusive capacity. Sodium intake has been shown to directly impact circulating SCFA levels (16, 52). Therefore, another limitation to the study is the lack of information regarding variations in inter-individual diets between patients at baseline. Obtaining and controlling for this data in future studies could help reduce the occurrence of confounding. Not having SCFA data from intracranial blood was not a limitation of the study as the indirect role of SCFAs were being observed, but having this data in future studies would allow for analysis of the direct role of SCFAs in the local cerebral environment. Lastly, as this study utilized many individual analyses among large data sets, the chance of false associations increases. Ideally, another study with data independent from the current one should be performed to verify the results found here. Future studies should aim to examine the gut microbiota in conjunction with plasma levels of SCFAs both at the time of stroke and longitudinally throughout stroke pathology to better establish an understanding of the transient changes in SCFA concentrations throughout the body and the subsequent function of these changes.

The current study provides novel data on plasma SCFAs taken at thrombectomy studied relative to stroke comorbidities and severity. Protein-protein networks provide insight into the potential molecular pathways that the SCFAs are both directly and indirectly affecting early in stroke and the clinical relevance associated with these interactions. Our results provide direction for future studies to look at how SCFAs change with gut dysbiosis as stroke pathology progresses and the clinical consequences of this. It also provides a framework for molecular studies to better characterize the intrinsic mechanisms of SCFAs. We conclude that the role of SCFAs and the effect of stroke on plasma SCFAs early in stroke warrants additional analysis.

## Data Availability Statement

The original contributions presented in the study are publicly available. This data can be found here: https://figshare.com/articles/dataset/BACTRAC_SCFA_Data_xlsx/17054096/1.

## Ethics Statement

The studies involving human participants were reviewed and approved by University of Kentucky IRB committee. The patients/participants provided their written informed consent to participate in this study.

## Author Contributions

NH wrote the manuscript, collected blood samples and performed data analysis. JF collected blood samples, analyzed data and edited the manuscript. CM was the statistician of the study. AT and AS edited the manuscript. AM aided in data analysis, manuscript editing and, with JC, was involved in SCFA quantification. JF edited the manuscript and was one of the neurosurgeons responsible for sample obtainment. KP edited the manuscript and oversaw the study and creation of the manuscript. All authors contributed to the article and approved the submitted version.

## Funding

Department of Neurology University of Kentucky.

## Conflict of Interest

KP, JFF, and AS are cofounders of Cerelux, LLC. JC is employed by Dicerna Pharmaceuticals Inc.

The remaining authors declare that the research was conducted in the absence of any commercial or financial relationships that could be construed as a potential conflict of interest.

## Publisher’s Note

All claims expressed in this article are solely those of the authors and do not necessarily represent those of their affiliated organizations, or those of the publisher, the editors and the reviewers. Any product that may be evaluated in this article, or claim that may be made by its manufacturer, is not guaranteed or endorsed by the publisher.
